# Rapid, Point-of-Care scFv-SERS Assay for Femtogram Level Detection of SARS-CoV-2

**DOI:** 10.1021/acssensors.1c02664

**Published:** 2022-03-10

**Authors:** Delphine Antoine, Moein Mohammadi, Madison Vitt, Julia Marie Dickie, Sharmin Sultana Jyoti, Maura A. Tilbury, Patrick A. Johnson, Karen E. Wawrousek, J. Gerard Wall

**Affiliations:** † Microbiology, College of Science and Engineering, and SFI Centre for Medical Devices (CÚRAM), National University of Ireland, Galway (NUI Galway), Galway H91 TK33, Ireland; ‡ Chemical Engineering, University of Wyoming, Laramie, Wyoming 82072, United States

**Keywords:** SARS-CoV-2, COVID-19, diagnostic, Raman spectroscopy, SERS, point-of-care, scFv antibody fragment

## Abstract

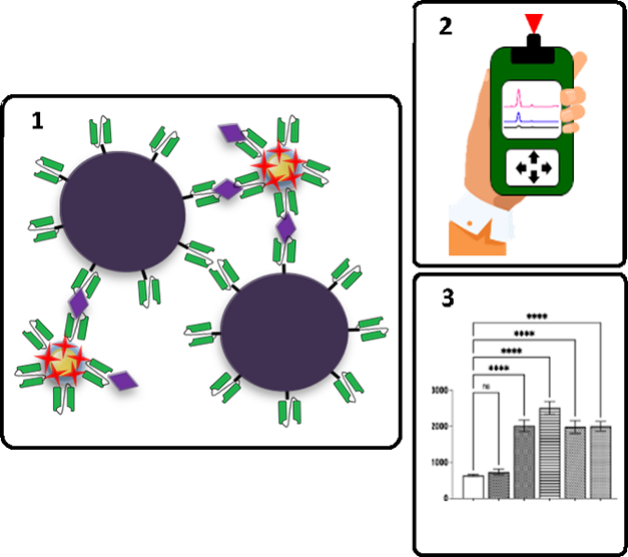

Rapid, sensitive, on-site identification of SARS-CoV-2 infections is an important tool in the control and management of COVID-19. We have developed a surface-enhanced Raman scattering (SERS) immunoassay for highly sensitive detection of SARS-CoV-2. Single-chain Fv (scFv) recombinant antibody fragments that bind the SARS-CoV-2 spike protein were isolated by biopanning a human scFv library. ScFvs were conjugated to magnetic nanoparticles and SERS nanotags, followed by immunocomplex formation and detection of the SARS-CoV-2 spike protein with a limit of detection of 257 fg/mL in 30 min in viral transport medium. The assay also detected B.1.1.7 (“alpha”), B.1.351 (“beta”), and B.1.617.2 (“delta”) spike proteins, while no cross-reactivity was observed with the common human coronavirus HKU1 spike protein. Inactivated whole SARS-CoV-2 virus was detected at 4.1 × 10^4^ genomes/mL, which was 10–100-fold lower than virus loads typical of infectious individuals. The assay exhibited higher sensitivity for SARS-CoV-2 than commercial lateral flow assays, was compatible with viral transport media and saliva, enabled rapid pivoting to detect new virus variants, and facilitated highly sensitive, point-of-care diagnosis of COVID-19 in clinical and public health settings.

The 2019 coronavirus disease (COVID-19), caused by severe acute respiratory syndrome coronavirus 2 (SARS-CoV-2), is classified as a global pandemic by the World Health Organization (WHO). Disease presentation varies from asymptomatic to severe outcomes, including potentially lethal respiratory tract infections,^1^ with clinical symptoms typically appearing within a few days of infection.^2^

The gold-standard method of SARS-CoV-2 identification is real-time reverse transcription quantitative polymerase chain reaction (RT-qPCR).^3,4^ RT-qPCR targets SARS-CoV-2-specific open reading frame 1ab (ORF1ab), nucleocapsid protein (N), envelope protein (E), or RNA-dependent RNA polymerase sequences,^3^ with typical limits of detection (LOD) around 10^3^ virus genomes/mL.^4^ While highly sensitive and specific, RT-qPCR is time-consuming, expensive, and requires qualified personnel and specialized equipment for RNA extraction and amplification.^5^ Furthermore, LODs vary considerably with the quality of extracted RNA and virus load at sampling.^5−7^

Enzyme-linked immunosorbent assays (ELISAs) have also been developed to detect SARS-CoV-2 and antivirus serological responses.^8,9^ Immunoassays exploit the specificity of antibodies to detect their target but lack the degree of amplification, which confers greater sensitivity on PCR-based approaches. While traditional ELISAs are laboratory-based, time-consuming (typically 3–4 h), expensive (particularly for antibody production), and require skilled personnel, point-of-care versions, such as lateral-flow (immuno)assays (LFA), overcome some of these limitations.^10^ LFAs generate results readable with the naked eye in a matter of minutes but with typical LODs of 10^5^ to 10^7^ genomes/mL,^11^ while they also suffer from quantification limitations and solid-phase effects.^12^

Surface-enhanced Raman scattering (SERS) spectroscopy is a powerful analytical tool based on a property of inelastic light scattering by Raman scatterers upon laser excitation, with scattering intensified 10^6^ to 10^11^-fold at or near the surface of noble metal substrates.^13^ SERS has been adapted to diagnostics by incorporating antibody moieties for biomarker recognition, leading to detection of viruses^14^ and antiviral IgGs at pg/mL concentrations.^15^ Most SERS immunoapproaches to date have utilized monoclonal or polyclonal antibodies that are laborious to isolate and expensive to produce.^14,16^

Numerous vaccines and antibody therapies developed to combat SARS-CoV-2 and reduce the severity of COVID-19 disease target the trimeric spike protein via which the virus gains entry to host cells.^17,18^ As mutations in the spike protein can lead to the emergence of virus variants with increased transmissibility, diagnostic tools that can rapidly and sensitively identify SARS-CoV-2, and quickly pivot to identify newly emerging virus variants, are a core facet in suppressing viral spread.

Antibody engineering provides a pathway to faster isolation (3–4 weeks) and less-expensive production of biorecognition moieties. Recombinant single-chain Fv (scFv) antibody fragments (antibody V_H_ and V_L_ domains, joined by a peptide linker^19^) retain the binding pocket of whole antibodies but lack constant domains, which are unnecessary *in vitro* and can cross-react in complex matrices.^20^ Importantly, due to their smaller size (27 kDa compared to 150 kDa monoclonal antibodies) and absence of glycosylation, scFvs can be expressed in inexpensive bacterial systems, such as *Escherichia coli*.^21^ Furthermore, large scFv libraries can be rapidly screened *in vitro* for molecules that bind a target antigen, without the need for immunization, via phage display technology.^22,23^

In the present study, we describe the isolation of scFvs that bind receptor-binding domain (RBD) of SARS-CoV-2 spike protein, and their incorporation into a point-of-care SERS immunoassay to detect SARS-CoV-2 using a commercially available, handheld, battery-operated device (Figure 1). The assay is sensitive to femtogram quantities of antigen and detects SARS-CoV-2 but does not cross-react with closely related human coronavirus HKU1 spike protein. The assay has potential for widespread use in rapid, point-of-care identification of SARS-CoV-2 infections.

**Figure 1:**
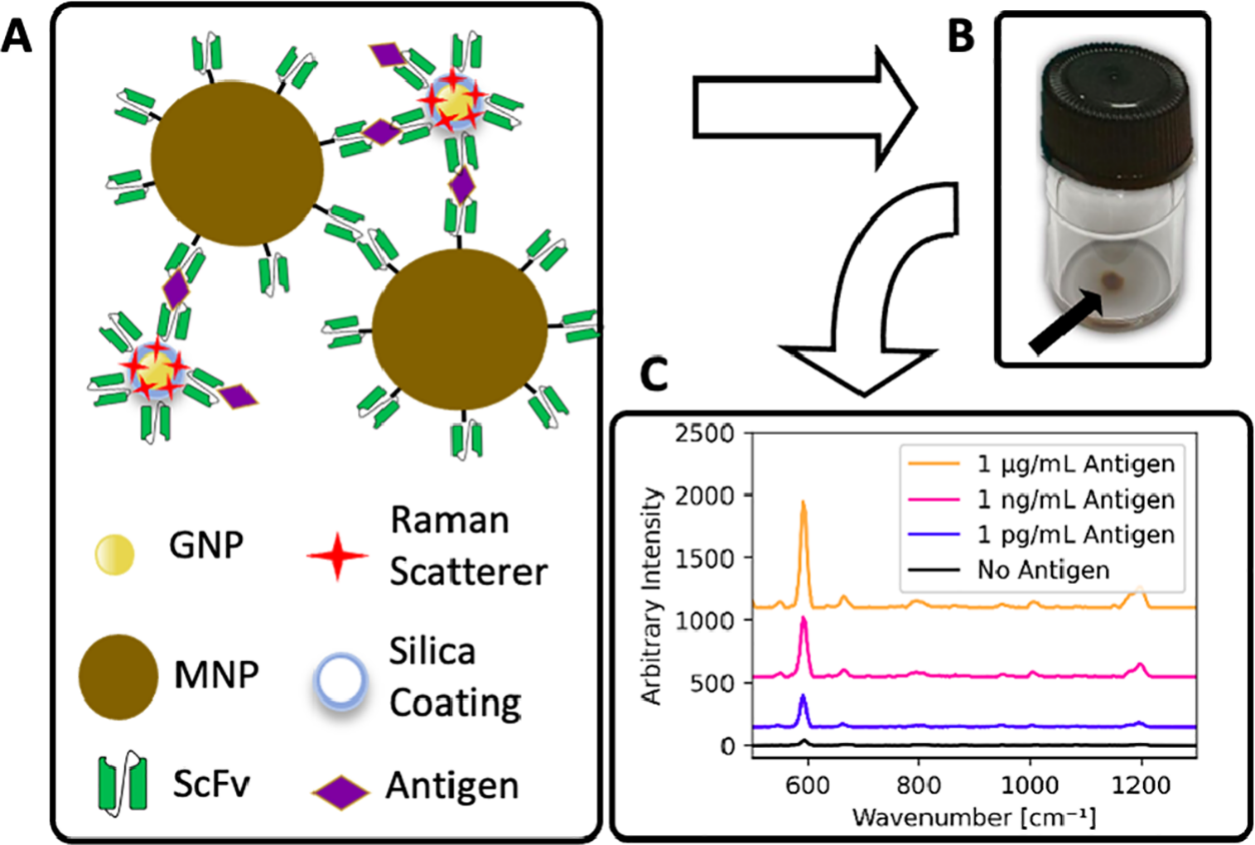
Schematic of the SERS immunoassay to detect SARS-CoV-2. (A) Magnetic nanoparticles (MNPs) and gold nanoparticles (GNPs) with a Raman scatterer, both coated with virus-binding scFv antibody fragments, form immune complexes in the presence of virus. (B) After pelleting the immunocomplexes (arrowed) using an external magnet, a handheld instrument is used to interrogate the pellet, (C) yielding a SERS spectrum that is diagnostic for the target.

## Experimental Section

### Materials

All chemicals were from Sigma-Aldrich unless otherwise specified. *E. coli* amber-suppressor strain TG1 was used to express phage-displayed scFvs for panning and to titer the library and helper phage. *E. coli* strain HB2151 was used for soluble expression of scFvs. The YamoI human scFv library was from Montarop Yamabhai.^22^ SARS-CoV-2 proteins and inactivated viruses from BEI Resources (Manasses, Virginia) are listed in the Acknowledgments.

### Selection of RBD-Binding ScFv-Phages

Procedures for phage library rescue and titration were as described in ref (22). The library was subjected to three rounds of panning on immunotubes (Maxisorb, Nunc) coated overnight at 4 °C with 200 μg (Round 1), 20 μg (Round 2), or 2 μg (Round 3) of RBD in 1 mL phosphate-buffered saline (PBS). After blocking (R1: Superblock (Thermo Fisher, Ireland); R2: 3% skimmed milk powder in PBS; and R3: 10% fetal bovine serum in PBS) for 2 h at room temperature, tubes were washed three times with PBS. Phage particles (10^12^ colony-forming units in 4 mL of the appropriate blocking solution) were added to tubes and incubated at room temperature for 1 h with rocking and 1 h without rocking. Unbound phages were removed by washing with PBS/Tween-20 (R1: eight washes with PBS/0.1% Tween-20; R2: R1 plus seven washes with PBS/0.2% Tween-20; and R3: R2 plus five washes with PBS/0.5% Tween-20), followed by 10–20 washes with PBS. Bound phages were eluted using 1 mg/mL trypsin for 10 min with rocking at room temperature, followed by 50 mM glycine-HCl (pH 2.0) for 10 min with rocking at room temperature, and neutralized using 200 mM NaHPO_4_, pH 7.5. Eluted phages were titered and rescued for successive panning rounds using the KM13 helper phage.^22^

### ELISA Analysis

RBD binding of polyclonal scFv-phage populations eluted after Rounds 1–3 and of monoclonal scFv-phage or soluble scFv isolated after Round 3 was assessed by ELISA (see the Supporting Information).

### Soluble ScFv Expression

Plasmid DNA was purified from individual *E. coli* TG1 clones expressing RBD-binding phages and used to transform *E. coli* HB2151 cells. Overnight *E. coli* cultures were recultured in 50 mL LB broth containing 100 μg/mL ampicillin to an OD_600_ of 0.6–0.7, when bacteria were induced with 1 mM isopropyl β-d-1-thiogalactopyranoside. Culture supernatants were collected after 20 h induction at 30 °C for scFvs 2, 3, and 37, followed by purification by immobilized metal affinity chromatography (IMAC). For scFv 10, cultures were induced at 20 °C, and periplasmic proteins were extracted using ice-cold TSE buffer (200 mM Tris, 500 mM sucrose, 1 mM ethylenediaminetetraacetic acid, pH 8.0) for 30 min on ice with rocking, followed by the addition of ice-cold distilled water for 1 h on ice with rocking. After centrifugation, the supernatant containing the soluble scFv was dialyzed overnight against 5 L of IMAC binding buffer (3.98 M NaCl, 80 mM NaH_2_PO_4_, 80 mM Na_2_HPO_4_·2H_2_O) at 4 °C.

### Protein Purification

For IMAC purification of scFvs, a 1 mL Hitrap column (GE Healthcare, UK) was loaded with Ni^2+^ before equilibration with wash buffer (20 mM sodium phosphate, 0.5 M NaCl, pH 7.4) containing 20 mM imidazole. Protein samples were filtered through a 0.4 μm filter, followed by addition of 20 mM imidazole before loading onto the column. The column was washed with 20, 30, and 10 mL wash buffer containing 20, 50, and 80 mM imidazole, respectively, and eluted using wash buffer containing 400 mM imidazole. Eluted fractions were dialyzed against PBS, and purified proteins were analyzed under denaturing conditions in 12% SDS-PAGE. Gels were stained with InstantBlue Coomassie stain (Expedeon, UK), or proteins were transferred to Amersham Protran 0.2 μm nitrocellulose blotting membrane (GE Healthcare, UK). Detection of scFvs utilized a monoclonal antipolyhistidine HRP-conjugated antibody diluted 1:1000 in PBS containing 1% bovine serum albumin (BSA). The color was developed using TMB.

### ScFv DNA Analysis

ScFv diversity was analyzed by *Bst*NI restriction analysis of plasmid DNA from RBD-binding clones and electrophoresis on 2% agarose. ScFv genes were sequenced at Eurofins Genomics (Ebersberg, Germany).

### SERS Nanotag Synthesis

SERS nanotags were generated by a layer-by-layer method. Briefly, 60 nm GNPs (Ted Pella, Redding, CA or synthesized in-house^24^) were labeled by mixing 2.6 × 10^10^ particles with 66 μL of fresh 0.8 mM Nile blue solution in a glass vial with stirring and sonication for 30 min. 1 mL of 4 g/L, pH 7.0 poly(acrylic acid) was added dropwise to stabilize Raman scatterers adsorbed onto GNPs, and the reaction was stirred for 3 h. Particles were washed twice with ultrapure water (16 000 × g, 10 min) and resuspended in 2 mL ultrapure water. A silica shell was then formed using a modified Stöber method.^25^ While stirring, freshly made 0.3 mM 3-aminopropyltrimethoxysilane (APTMS) was added at a ratio of 1 μL of APTMS to 1 mL of coated GNPs, and after 20 min, 17 mL of 2-propanol and 200 μL of EMSURE ammonia solution (25%) were added under gentle stirring. Tetraethyl orthosilicate (TEOS) was added in four portions (0.5 μL every 15 min) with gentle stirring for an additional 16 h. Excess silica was removed by two absolute ethanol washes (4415 × g, 15 min), and particles were resuspended in 3 mL absolute ethanol. To functionalize silica-coated particles with thiol groups, 1 μL of 0.3 mM degassed 3-mercaptopropyltriethoxysilane (MPTES) was mixed with 1 mL of particles, followed by incubation for 6 h at 37 °C with shaking at 170 rpm. Particles were washed twice with absolute ethanol (16 000 × g, 10 min) and twice with ultrapure water.

### ScFv Conjugation to Particles

ScFvs were conjugated to 10 mg/mL magnetic particles (Pierce NHS-Activated Magnetic Beads, cat. #88826, Invitrogen) following the manufacturer’s protocol (see Supporting Information).

### SERS Immunoassay

SERS immunoassays were conducted by combining 3 μL scFv-conjugated magnetic beads (2.88 × 10^7^ particles), 12 μL scFv-conjugated SERS nanotags (6.24 × 10^9^ particles), the desired amount of antigen prepared as a mock 50 μL sample in viral transport medium (VTM), and reaction buffer (1× PBS, 1% BSA) to a final volume of 500 μL in a 2 mL glass vial (cat #67502010, Metrohm, Laramie, WY). Assays were incubated at room temperature, shaking at 200 rpm for 20 min, after which formed immunocomplexes were pelleted using an external magnet. Raman spectra of pellets with solution on top were measured with a Mira DS handheld Raman spectrometer (Metrohm) with O-Ring 41 (part #4011953, Danco) inserted into the vial holder to focus the 785 nm laser on the pellet. Spectra were collected using a laser power of 5 (50 mW), integration time 1 s, and raster off, and the peak height was measured at 591 cm^–1^. All assays were performed in triplicate, and all concentrations are reported as the antigen concentration in 50 μL of VTM.

### Limit of Detection Calculations

Varying concentrations of purified Wuhan-Hu-1 trimeric spike protein and inactivated Wuhan-Hu-1 SARS-CoV-2 were used to determine LOD. Signal intensities for LOD were calculated using LOD = *X* + 3SD, where *X* is the mean and SD is the standard deviation of the negative control.^26,27^ These values were 718 and 4343 for the spike protein and inactivated virus, respectively. Antigen concentration versus Raman intensity was graphed, and logarithmic fits for the points above the mentioned LOD intensities were used to convert the Raman intensity at the LOD to an antigen concentration. Equations used were *y* = 292.81ln(*x*) – 906.54 and *y* = 431.96 ln(*x*) – 244.43 for spike protein and inactivated virus, respectively.

## Results and Discussion

### Isolation of RBD-Binding ScFvs

Phage display technology is a powerful tool for *in vitro* isolation of high-affinity antibody fragments against ligands of interest. Compared to traditional, immunization-based methods, it is robust, cost-effective, relatively simple to carry out, does not require animals, and yields target-binding antibody molecules in weeks rather than months.^22,23^

The YamoI scFv library, which contains more than 10^8^ different human scFvs generated from 140 non-immunized donors,^22^ was subjected to biopanning against immobilized RBD: the domain of the homotrimeric spike protein via which SARS-CoV-2 interacts with human ACE2 to effect cell entry.^28^ RBD was selected as a diagnostic target due to its high expression level on SARS-CoV-2,^29^ for increased detection sensitivity, and its relatively poorly conserved sequence amongst human coronaviruses,^17^ to provide specificity for SARS-CoV-2.

**Figure 2:**
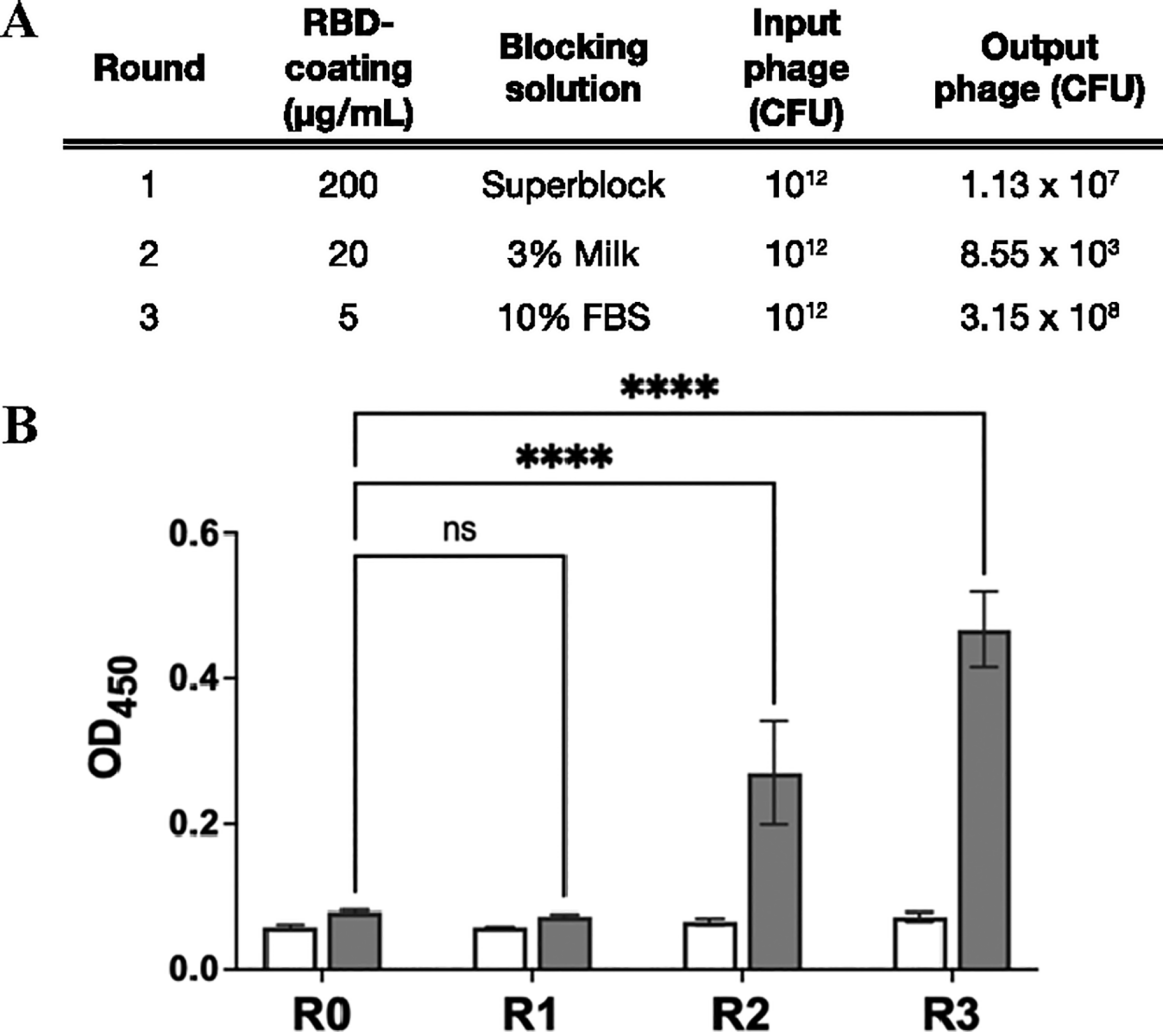
Isolation of RBD-binding scFvs by phage display. (A) Titers of input and output phage populations throughout the three rounds of library panning. CFU = colony-forming units. (B) ELISA analysis of RBD binding of eluted polyclonal phage-scFv populations. R0: unpanned library; R1–R3: polyclonal phage populations eluted after rounds 1–3 of library panning against immobilized RBD. ELISA wells were coated with blocking solution (light columns) or 2 μg/mL SARS-CoV-2 Wuhan-Hu-1 RBD (dark columns). Values represent the average of three replicate wells, and error bars indicate the standard deviation. A one-way ANOVA, followed by Dunnett’s multiple comparisons test was performed: ns = not significant; ****: *p* < 0.0001.

Increasing washing stringency, decreasing RBD concentration, and varying blocking solution were utilized through three rounds of biopanning to isolate RBD-binding scFvs (Figure 2A), which was evidenced by increased RBD binding of polyclonal phage populations eluted after the second and third biopanning rounds (Figure 2B).

### Analysis of Anti-SARS-CoV-2 ScFvs

**Figure 3:**
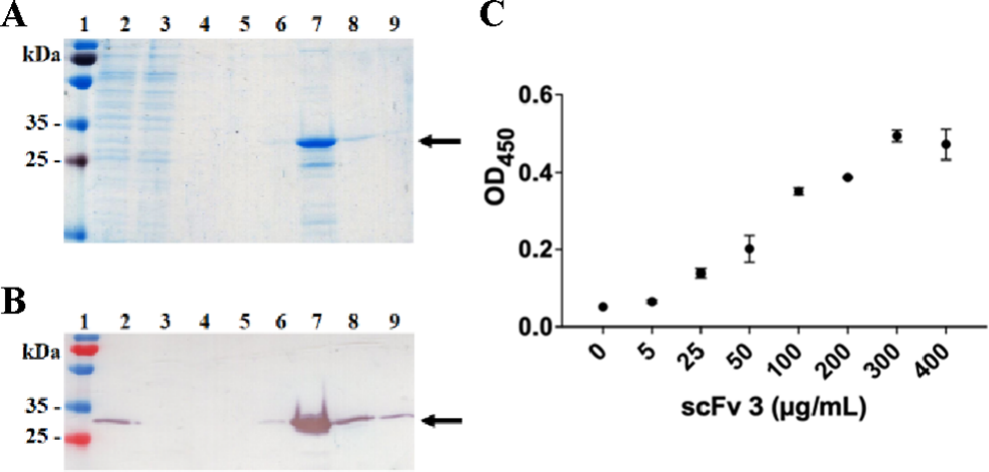
Purification and SARS-CoV-2 Wuhan-Hu-1 spike protein binding of scFv3 expressed in *E. coli*. (A) Coomassie-stained SDS-PAGE and (B) Western blot analysis of scFv3 purification. 1: molecular weight markers; 2: *E. coli* culture supernatant; 3: IMAC column flow through; 4–5: column washes with 20 and 50 mM imidazole, respectively; and 6–9: fractions eluted using 400 mM imidazole. Arrows indicate the expected molecular weight. (C) ELISA analysis of binding of purified scFv3 to 4 μg/mL SARS-CoV-2 Wuhan-Hu-1 spike protein. Values represent the average of three replicate wells, and error bars indicate the standard deviation.

To isolate individual RBD-binding antibody fragments from the polyclonal eluates, phage-displayed scFvs from 40 randomly selected *E. coli* clones from panning round R3 were screened by ELISA. This identified nine RBD-binding scFvs, DNA fingerprinting of which revealed four distinct profiles. DNA sequencing established the sequences of the four full-length scFv genes, encoding scFvs 2, 3, 10, and 37. The four scFvs were expressed in a soluble, non-phage-linked format using the non-amber-suppressor *E. coli* HB2151 strain, and purified at yields from 0.5 to 2 mg scFv per liter of bacterial culture (data shown for scFv3 in Figure 3). Half maximal effective concentrations (EC_50_)—a measure of antibody binding used to screen and affinity-rank antibody molecules^30^—of 12, 182, and 86 μg/mL were determined by ELISA for RBD for scFvs 2, 3, and 37, respectively (Figure S1). The EC_50_ of scFv10 could not be calculated as signal saturation was not achieved at 350 μg/mL scFv. As scFvs were isolated against the RBD of spike protein, the ability of scFv3 to bind full-length spike protein was also confirmed (Figure 3C), with an EC_50_ of 81 μg/mL.

ScFv production in *E. coli* provides a cost-effective alternative to mammalian expression of whole antibodies or the use of commercial antibodies in immunosensor development, as it is faster, less expensive, generates higher yields, and is easier to scale up than mammalian cell expression.^19^ While *E. coli* is poorly adapted to produce large, glycosylated immunoglobulins, it is ideally equipped to express smaller antibody-derived fragments such as scFvs.^19,31^ ScFvs are also particularly suited to *in vitro* applications as their variable domains provide binding specificity similar to whole antibodies but the absence of constant domains—necessary *in vivo* for immune effector functions—reduces the potential for cross-reactivities. Meanwhile, their smaller size and more compact shape^19,20^ allow for denser packing of target recognition sites on sensor surfaces for improved detection.

### SERS Immunodetection of Viral Antigen

While SERS immunoassays share antigen recognition and immunocomplex formation with other antibody-based approaches, SERS detection is orders of magnitude more sensitive than colorimetric or fluorescence-based ELISA, or lateral flow methods.^15,32^ The SERS nanotags used in this study consist of a 56.6 ± 9.0 nm gold core to which a Raman-scattering dye, Nile blue, was adsorbed and encapsulated within a 5.4 ± 2.1 nm silica shell (Figure S2). Nile blue is a redox dye with a characteristic Raman peak observed at 591 cm^–1^ (Figure S3). The assay was established based on our previous studies using whole antibodies,^14,16^ with the incubation time with antigen (20 min) and collection times with the magnet (10 min) varied to optimize the signal to background ratio for the SERS-scFv assay (Figure S4). Assays were carried out in VTM as this is the typical storage medium for SARS-CoV-2 swabs.

**Figure 4:**
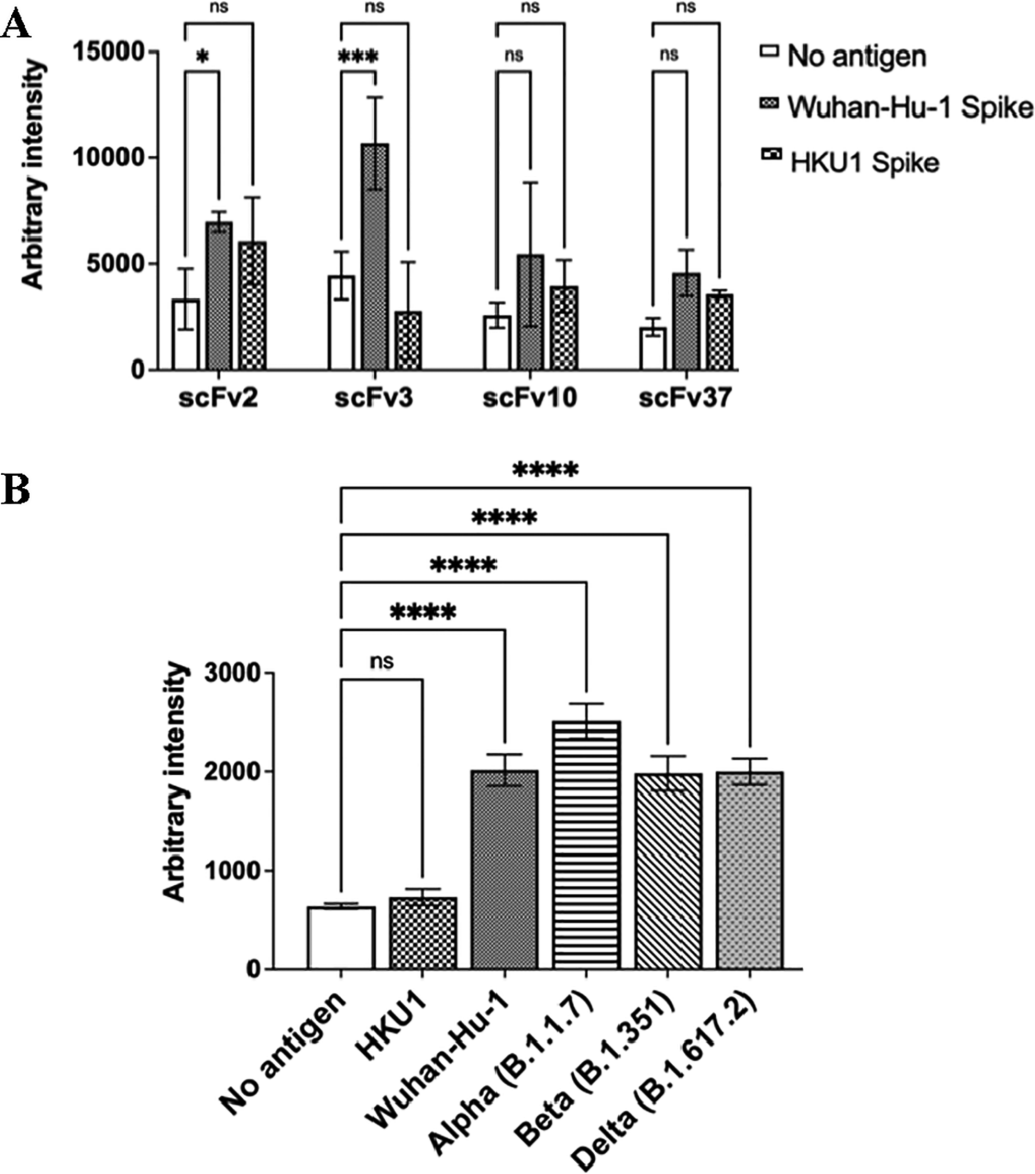
ScFv specificity for SARS-CoV-2 spike proteins. (A) SERS assays were performed with the same scFv on both MNP and SERS nanotags. Reactions contained no antigen or 50 ng of Wuhan-Hu-1 SARS-CoV-2 or HKU1 coronavirus homotrimeric spike protein. (B) SERS assays with scFv3 on both MNP and SERS nanotags (scFv3+3 assay). Assays were performed with no antigen or 100 pg of the relevant spike protein: HKU1, Wuhan-Hu-1 SARS-CoV-2, and alpha (B.1.1.7), beta (B.1.351), and delta (B.1.617.2) variants. Data are an average of three replicates and error bars indicate the standard deviation. Two-way (A) or one-way (B) ANOVA by Dunnett’s multiple comparisons tests were performed: ns = not significant; *: *p* < 0.05; ***p < 0.001; and ****p < 0.0001.

SARS-CoV-2 exhibits high overall sequence identity with other beta viruses of the Coronaviridae family, including 81% with HKU1, 80% with SARS-CoV, and 50% with MERS-CoV.^33^ Spike protein sequences are less conserved between the viruses,^17^ however, potentially allowing discrimination between SARS-CoV-2 and HKU1, which is responsible for common cold infections. Of the four scFvs, scFv3 strongly differentiated between SARS-CoV-2 and HKU1 coronavirus homotrimeric spike proteins in assays utilizing the same scFv tethered to the MNP and SERS nanotags (Figure 4A). Additional assays containing only scFv3 (scFv3+3 assays) carried out with 1000× higher concentrations of HKU1 spike protein yielded no significant signal (Figure S5), indicating that common coronavirus infections are unlikely to cause false-positive results in the assay.

The sensitivity of SERS immunoassays combining scFv3 with scFvs 2, 10, or 37 (scFv3 on SERS nanotags and scFv 2, 10, or 37 on MNPs) was compared to that the scFv3+3 assay. After incubation with 50 ng of Wuhan-Hu-1 S trimer, the signal/background ratio for the scFv3+3 assay was 2.4, and only 1.2, 1.7, and 1.8 for the scFv3+2, 3 + 10, and 3 + 37 reactions, respectively (data not shown). Therefore, based on its sensitivity for SARS-CoV-2 spike protein and lack of cross-reactivity with HKU1 protein, the scFv3+3 SERS immunoassay was utilized in further validation studies.

As mutations in the antibody target can compromise diagnostic tests,^34^ we investigated the ability of the scFv3+3 assay to detect SARS-CoV-2 variants with known RBD mutations. Homotrimeric spike proteins from all three lineages investigated were successfully detected, with signal intensity equal to or greater than those of the Wuhan-Hu-1 strain: B.1.1.7 (“alpha”: N501Y mutation in RBD), B.1.351 (“beta”: K417N/E484K/N501Y), and B.1.617.2 (“delta”: L452R/T478K)^35^ (Figure 4B). As further virus variants emerge, the speed and economy of the outlined biopanning-scFv expression-SERS assay pipeline, compared to traditional antibody isolation and production approaches,^19^ will enable diagnostic tests to be produced and validated in a matter of weeks, for continued monitoring and control of evolving outbreaks. Additionally, the multiplex capability of SERS tests, exploiting the non-overlapping Raman peaks of different scatterers,^16^ creates the potential to develop an assay utilizing multiple scFvs, which can distinguish between virus variants in a rapid, on-site assay.

### Detection of Virus

As scFvs were isolated based on their binding of purified RBD, the ability of the scFv3+3 SERS immunoassay to detect whole virus was investigated. Gamma-inactivated SARS-CoV-2 USA-WA1/2020 isolate, raised in Vero cells, and heat-inactivated SARS-CoV-2 B.1.1.7 strain (alpha variant), cultured in Vero E6 cells, were used at a concentration of 6.49 × 10^5^ genomes/assay, the equivalent of 5–19 pg of purified spike protein.^29^ All viruses were detected with a signal similar to the 5 pg Wuhan-Hu-1 spike protein positive control (Figure S6). No difference between detection of heat-inactivated and gamma-inactivated virus SARS-CoV-2 Wuhan-Hu-1 virus was observed.

### Limit of Detection of SERS Immunoassay

**Figure 5:**
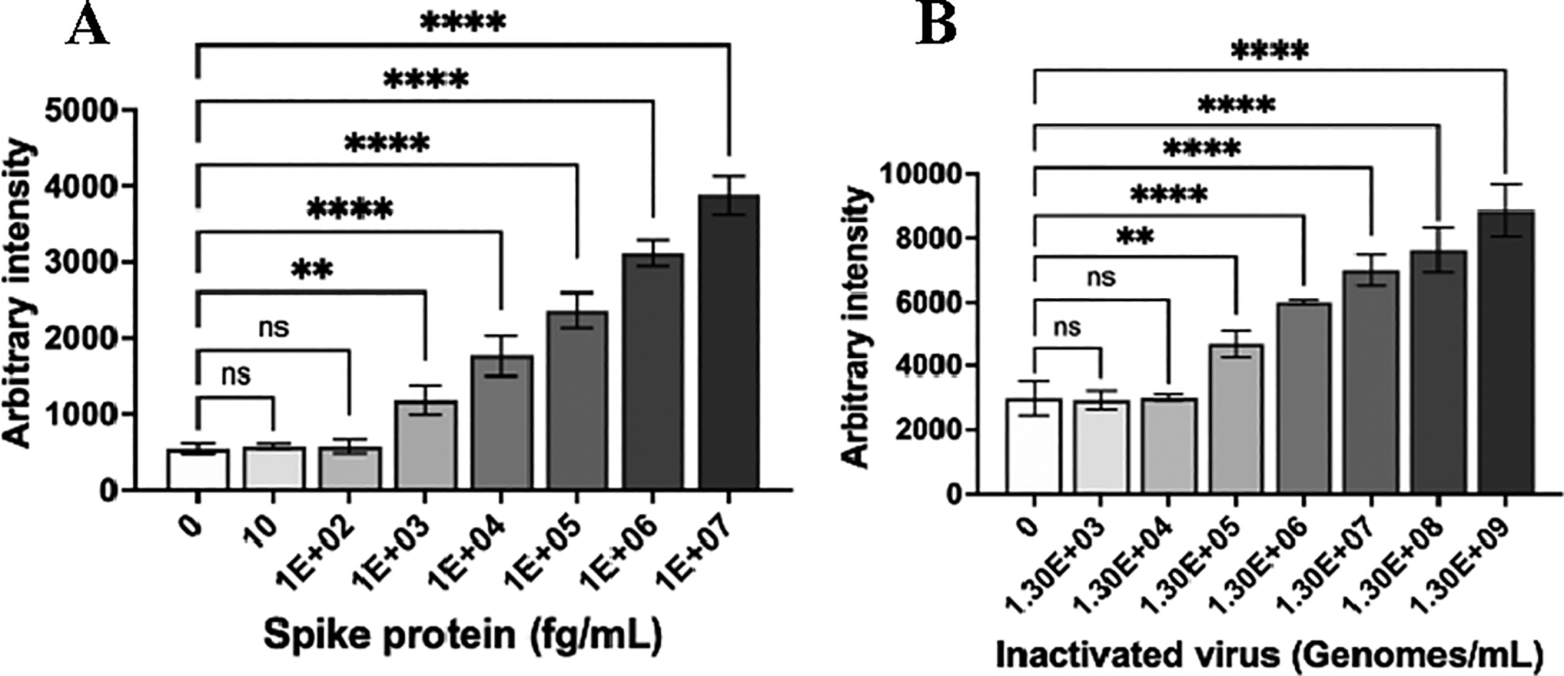
Limit of detection of scFv3+3 SERS assay for: (A) purified SARS-CoV-2 Wuhan-Hu-1 spike protein or (B) gamma-irradiated SARS-CoV-2 to simulate a diagnostic sample. Signals are reported as an average of three replicates, and error bars represent the standard deviation. A one-way ANOVA, followed by Dunnett’s multiple comparison test was performed: ns = not significant; **: *p* < 0.01; ****: *p* < 0.0001.^46^

The LOD of the scFv3+3 SERS assay was determined using purified Wuhan-Hu-1 spike protein and inactivated Wuhan-Hu-1 SARS-CoV-2 (Figure 5). The LOD for spike protein was calculated as 257 fg/mL and the LOD for inactivated virus as 4.1 × 10^4^ genomes/mL in the mock sample (Figure S7). This sensitivity compares favorably with immunoassays and lateral flow COVID-19 tests, which have typical reported LODs in the ng/mL or pg/mL range,^36−39^ or 10^5^–10^7^ genomes/mL in the case of LFAs.^11^ In this study, the LOD for gamma-inactivated SARS-CoV-2 Wuhan-Hu-1 virus was demonstrated to be between 6.56 × 10^5^ and 1.31 × 10^6^ genomes/mL using a commercial LFA (Figure S8)—corresponding to 16× to 32× less sensitive than the scFv3+3 SERS immunoassay. Meanwhile, we have previously demonstrated a SERS assay to be >400-fold more sensitive than an ELISA using the same reagents.^15^

The extremely high sensitivity of the scFv3+3 assay derives from the ability of the SERS platform to differentiate Raman peaks from the background signal at very low target concentrations.^32^ Multiple studies have reported that viral loads in infected individuals typically peak around symptom onset or within 2–4 days, followed by a gradual decrease over the next 1–3 weeks.^40,41^ Virus loads of 1.5 × 10^6^ and 1.6 × 10^5^ virus copies/mL have been measured in respiratory tract specimens from patients upon presentation with severe and mild COVID-19 disease, respectively.^42^ The on-site detection of virus at the LOD of 4.1 × 10^4^ genomes/mL in 30 min by the present test, therefore, suggests a clear public health utility that complements existing test formats. Importantly, while the assay was developed for VTM, a preliminary investigation indicated its compatibility with saliva samples also (Figure S9). Additionally, the assay requires minimal power, relying just on a magnetic separation step and a battery-operated spectrometer.

Most approved RT-qPCRs have LODs around 10^3^ RNA copies per mL,^43^ with a range from 180 to 600 000 “detectable units” (effectively virus genomes) per mL amongst tests with FDA Emergency Use Authorizations.^44^ Patient samples exhibiting fewer than 10^6^ copies of E or N protein mRNA per mL have been reported to contain minimal or no measurable infectious virus;^41^ this has been proposed to indicate that RT-qPCR detects trace RNA amounts after the virus is no longer transmissible, particularly at high cycle threshold values.^43,45^ Importantly also, as RT-qPCR requires sample transport to dedicated laboratories for RNA purification, amplification, and analysis, its test-to-result time is typically at least 1–2 days, which critically reduces the efficacy of testing and of control of virus spread, compared with rapid, point-of-care tests such as the present assay.^43,45^

## Conclusions

The scFv-SERS detection assay described offers highly sensitive virus detection, a rapid sample-to-result time (30 min), and point-of-care useability, using a commercial, hand-held Raman instrument. It has an LOD of 257 fg/mL spike protein or 4.1 × 10^4^ virus genomes/mL in VTM, which is 1–2 orders of magnitude lower than virus loads typical of infectious individuals. The assay detects B.1.1.7, B.1.351, and B.1.617.2 virus variant spike proteins but does not cross-react with common coronavirus HKU1 spike protein. Its use of *in vitro* scFv isolation and *E. coli* expression technologies enables a fast pivot to detect newly emergent variants. The assay can be used to support widespread, sensitive identification of SARS-CoV-2 in clinical, public health, and point-of-care settings.

## Supporting Information Available

Experimental method for ELISA analysis of scFvs and scFv conjugation to particles; EC_50_ data for RBD-binding scFvs; experimental method and TEM image of SERS nanotags; structure and Raman spectrum of Nile blue; optimization of SERS assay incubations; detection of Wuhan-Hu-1 and HKU1 spike proteins; detection of inactivated SARS-CoV-2 virus variants; calculation of LODs for spike protein and virus; experimental method and lateral flow assay detection of inactivated SARS-CoV-2; and comparison of detection of SARS-CoV-2 in PBS, VTM, and saliva(PDF)

## Notes

D.A. and M.M. contributed equally. The manuscript was written through contributions of all authors. All authors have given approval to the final version of the manuscript.
