# Supplementary material for: Rapid, Point-of-Care scFv-SERS Assay for Femtogram Level Detection of SARS-CoV-2
Source: ACS Sens. 2022 Mar 10;7(3):866–73. doi: 10.1021/acssensors.1c02664 (PMC8961876; doi:10.1021/acssensors.1c02664)
Supplement: Supplementary file 1 — se1c02664_si_001.pdf [file se1c02664_si_001.pdf]

A rapid, point-of-care scFv-SERS assay for femtogram level detection of SARS-CoV-2

Delphine Antoine[#,‡], Moein Mohammadi[§,‡], Madison Vitt[§], Julia Marie Dickie[§], Sharmin Sultana Jyoti[§], Maura A. Tilbury[#], Patrick A. Johnson[§], Karen E. Wawrousek[§,*], and J. Gerard Wall[#,*]

[#] Microbiology, College of Science and Engineering, and SFI Centre for Medical Devices (CÚRAM), National University of Ireland, Galway (NUI Galway), Galway H91 TK33, Ireland

[§] Chemical Engineering, University of Wyoming, Laramie, WY 82072, USA

Supporting Information for Publication

Materials and Methods

ELISA Analysis

RBD-binding of polyclonal scFv-phage populations eluted after Rounds 1-3 and of monoclonal scFv-phage or soluble scFv isolated after Round 3 was assessed by ELISA. Wells of a 96-well microtiter plate (Maxisorb, Nunc) were coated overnight at 4 °C with 2 μg/mL of RBD (Sino Biological, China) or 4 μg/mL of Wuhan-Hu-1 spike protein (BEI Resources) in PBS. After 3 washes with PBS, wells were blocked (phage particles from Round 1: 3% skimmed milk powder; Round 2: 3% ovalbumin; Round 3: Superblock, Thermo Fisher) for 2 h at room temperature. After five washes with PBS, wells were incubated with 100 μL of scFv-phages or soluble scFvs for 1 h at room temperature. Wells were washed three times with PBS/0.1% Tween-20 and three times with PBS, followed by 1 h incubation at room temperature with anti-M13 horseradish peroxidase (HRP)-conjugated IgG at 0.4 μg/mL in PBS/1% bovine serum albumin (BSA) for scFv-phage analysis, or an anti-c-*myc* HRP-conjugated IgG (Abcam, UK), diluted 1:250 in PBS/1% BSA, for soluble scFv analysis. After repeating the wash step, 100 μL of 3,3',5,5'-tetramethylbenzidine (TMB) substrate was added, reactions were stopped using 100 μL 1 M H_2SO_4, and absorbances were read at OD_{450}. Half maximal effective concentrations (EC_{50}) were calculated with GraphPad Prism Version 8.1, fitting to a five-parameter logistic curve.

ScFv Conjugation to Particles

ScFvs were conjugated to 10 mg/mL magnetic particles (Pierce NHS-Activated Magnetic Beads, cat. #88826, Invitrogen) following the manufacturer's protocol. To activate scFvs to bind thiol groups on SERS nanotags, 25 μL sulfo-SMCC (0.4 mg/mL) was mixed with 100 μg scFv for 45 min with shaking at 200 RPM, and buffer-exchanged with ultrapure water using a 2 mL 1000 Da MWCO Zeba spin column (Thermo Fisher). Activated scFvs were combined with 1 mL of 1 mg/mL SERS nanotags, incubated for 3 h at room temperature with gentle shaking, and the reaction was quenched with 10 μL sodium 2-mercaptoethanesulfonate (MESA) for 45 min. ScFv-conjugated nanotags were rinsed (1,000 x g, 7 min) once with 0.5X PBS and thrice with 0.5X PBS, 1% BSA, sonicating between washes. Particles were resuspended in 1 mL 0.5X PBS, 1% BSA, and stored at 4 °C. Conjugated scFvs were quantified by Micro BCA assay (Thermo Scientific), with particle removal prior to spectroscopic measurement. The average scFv concentrations on magnetic particles and SERS nanotags were 82 μg/mL and 26 μg/mL, respectively.

Transmission Electron Microscopy

To image SERS nanotags, the particles were air dried on a pure carbon 400 mesh Cu grid (Ted Pella) and bright field images collected with an FEI TECNAI G2 F20 S-Twin Transmission Electron Microscope (TEM; FEI, Hillsboro, OR) at the HRTRM facility, University of Wyoming. Average particle diameter and thickness of the silica shell were calculated after image analysis of 134 particles in ImageJ[46].

Lateral Flow Assays

Gamma-irradiated Wuhan-Hu-1 SARS-CoV-2 was diluted in the provided buffer and applied to BinaxNOW™ COVID-19 Ag CARD (Abbott) rapid LFA tests. Tests were carried out according to the manufacturer's instructions, with 250 μL of virus suspension utilized to represent six drops. VTM is not recommended for use with the test.

Figures

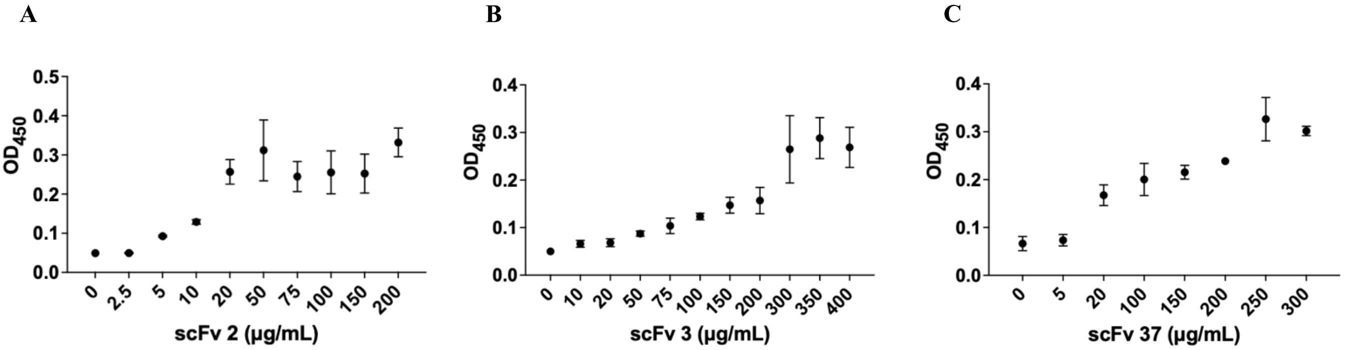

Figure S1. ELISA analysis of binding of purified scFv 2 (A), scFv 3 (B), and scFv 37 (C) to 2 µg/mL SARS-CoV-2 Wuhan-Hu-1 RBD protein. Values represent the average of three replicate wells, and error bars indicate the standard deviation.

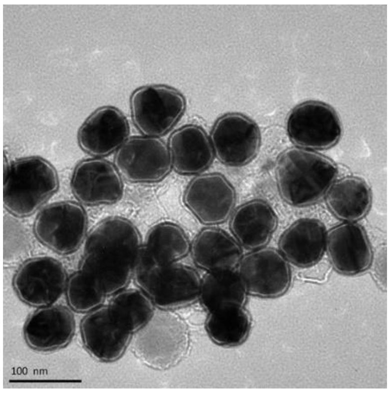

Figure S2. TEM of SERS nanotags with silica shell.

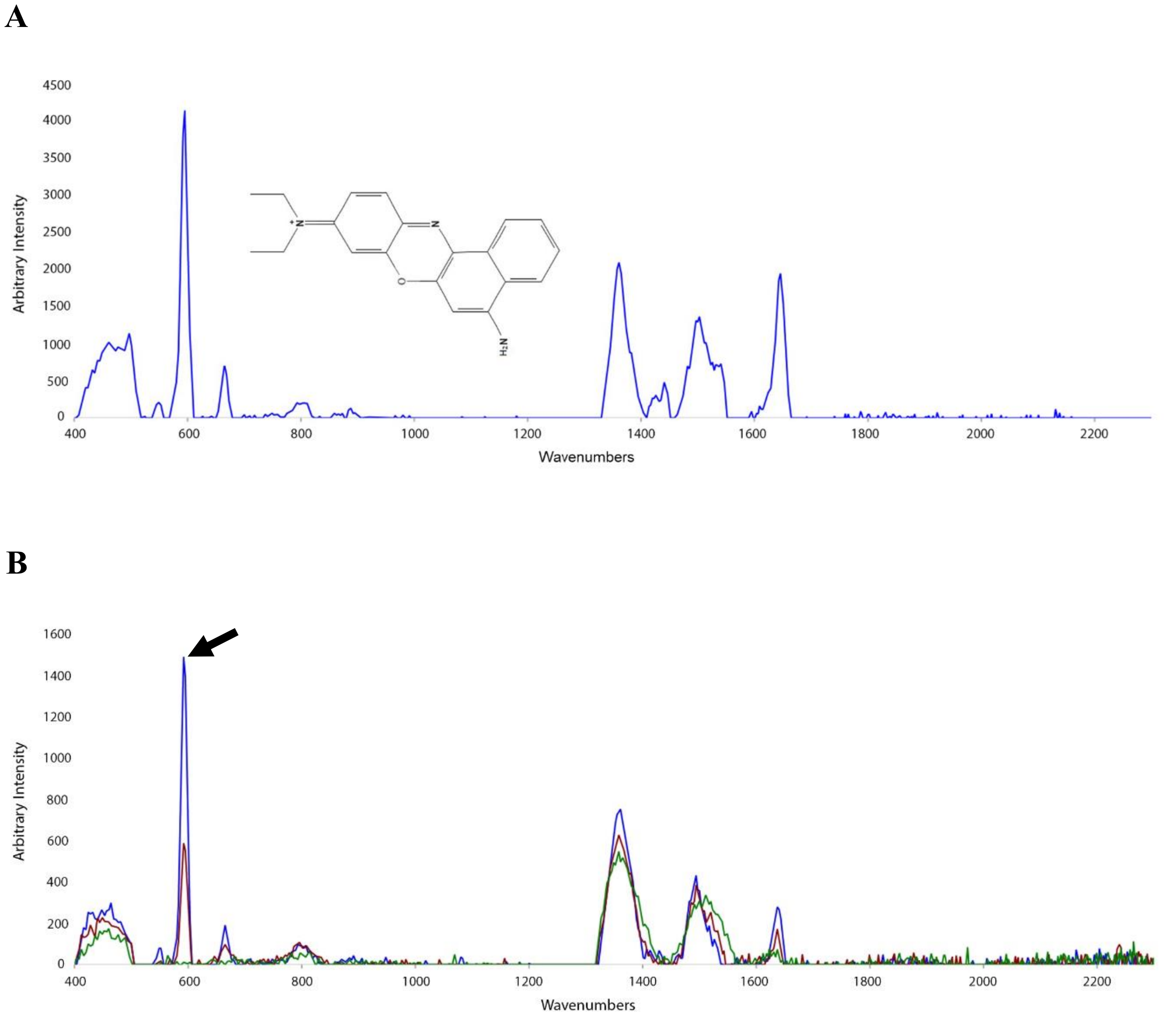

Figure S3. (A) Raman spectrum of Nile blue, which was used as Raman reporter in the study, with (inset) Nile blue structure. The spectrum was measured in a glass vial. (B) Experimental SERS spectra measured for reaction buffer (10% VTM in PBS; green), negative control (scFv3+3 SERS assay carried out in the absence of antigen; red spectrum), and positive sample (scFv3+3 SERS assay carried out in the presence of 50 ng of Wuhan-Hu-1 trimeric spike protein; blue spectrum). All reactions were carried out and spectra measured in glass vials. The strong signature peak of Nile blue at 591 cm^{-1}, which is due to the in-plane deformation of the heterocyclic ring, is indicated by the arrow.

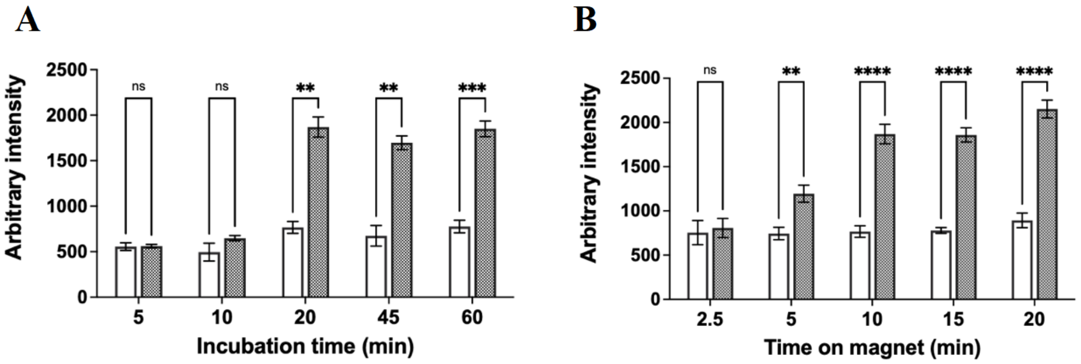

Figure S4. Optimization of scFv3+3 SERS assay to detect SARS-CoV-2. Signal/background ratio of SERS signal is shown with (A) varying times of incubation with antigen, and (B) varying collection times on an external magnet. SERS assays contained scFv3 on both MNP and SERS nanotags, and 50 ng of Wuhan-Hu-1 S trimer was used as antigen. Signal/Background ratios were calculated by dividing the SERS signal in the presence of antigen (shaded bars) by the signal in the absence of antigen (white bars). Assays were performed in triplicate; error bars indicate the standard deviation. For statistical analysis, two-way ANOVA followed by Šidák's multiple comparisons test was performed: ns = not significant. **: $p<0.01$; ***: $p<0.001$; ****: $p<0.0001$.

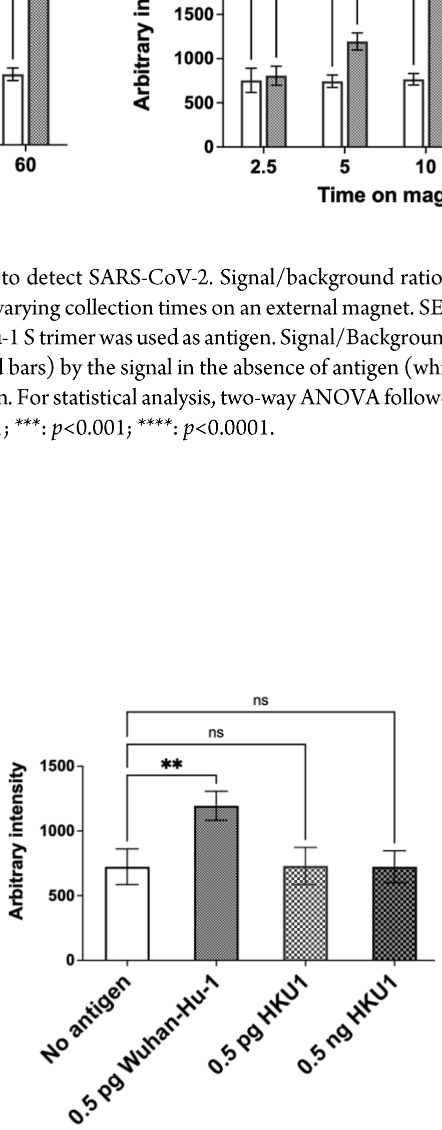

Figure S5. HKU1 spike protein in scFv3+3 SERS assay. SERS signals with varying concentrations of HKU1 spike protein were measured, with comparison to a negative control with no protein and a positive control containing 0.5 pg of Wuhan-Hu-1 spike protein. Data is an average of three replicates and error bars indicate the standard deviation. For statistical analysis, one-way ANOVA followed by Dunnett's multiple comparisons test was performed: ns = not significant. **: $p<0.01$.

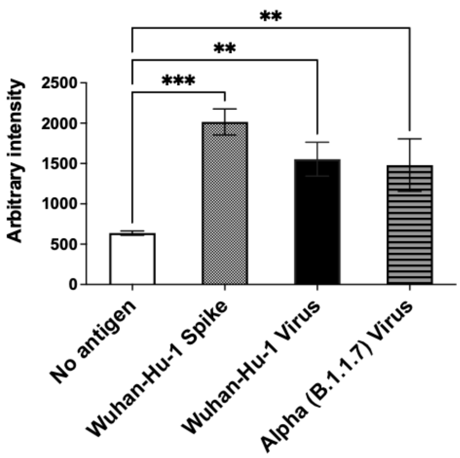

Figure S6. ScFv specificity for SARS-CoV-2 viruses in a SERS assay. ScFv3+3 SERS assays with gamma-irradiated SARS-CoV-2 isolate USA-WA1/2020 and heat-inactivated SARS-CoV-2 B.1.1.7 virus (alpha variant). All viruses were present at $6.49x10^5$ genome copies. Negative control contained no antigen and 5 pg of trimeric Wuhan-Hu-1 Spike protein served as a positive control. Data is an average of three replicates and error bars indicate the standard deviation. For statistical analysis, one-way ANOVA followed by Dunnett's multiple comparisons test was performed: ns = not significant. **: $p<0.01$; ***: $p<0.001$.

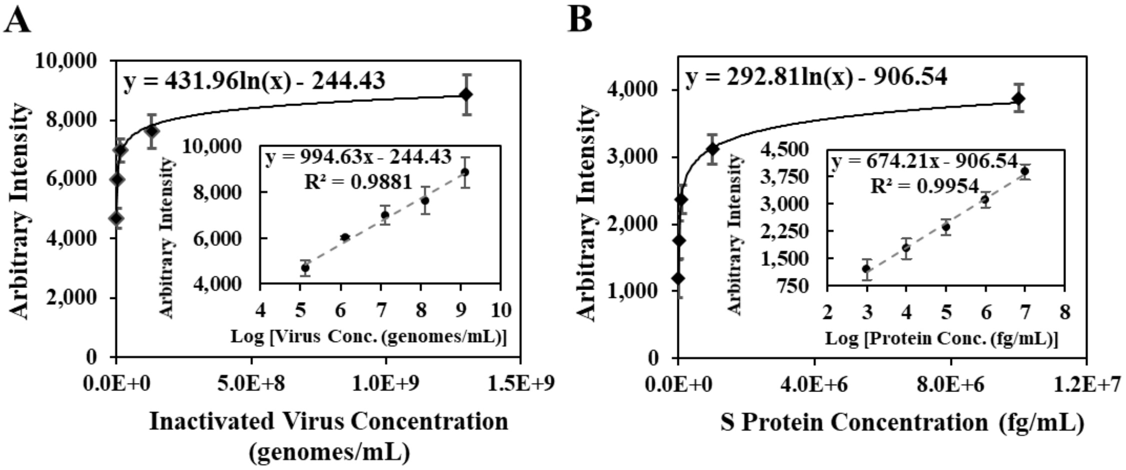

Figure S7. SERS signal with varying antigen concentration. SERS signals from scFv3+3 assays with varying concentration of (A) gamma-irradiated SARS-CoV-2 isolate USA-WA1/2020 or (B) Wuhan-Hu-1 S protein. Data is an average of three replicates and error bars indicate the standard deviation. Semi-log plots were plotted, and a log trend line was plotted and used to convert the signal intensity calculated for the limit of detection to determine LOD concentrations. To evaluate the fit of that trendline using R^2, a measure of linear regression, signal intensity was plotted against the log of the antigen concentration, as this is a linear relationship. The R^2 of the trendline correlating signal intensity to the log of S protein concentration is 0.9954, while that correlating signal intensity to the log of inactivated virus concentration is 0.9881, both indicating a good fit of the trendline to the data. Data points and equations are valid only above the limit of detection (LOD).

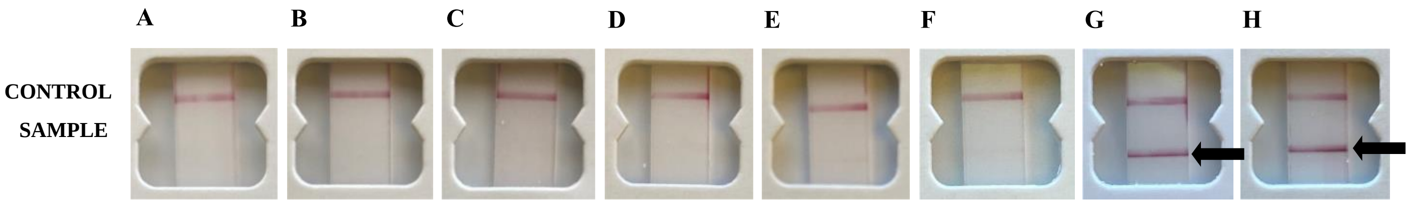

Figure S8. Lateral flow assay detection of inactivated SARS-CoV-2 using BinaxNOW™ COVID-19 Ag CARD (Abbott) rapid LFA tests. Virus concentrations are (A) 0, (B) 4.1x10^4, (C) 8.2x10^4, (D) 1.64 x10^5, (E) 3.28 x10^5, (F) 6.56 x10^5, (G) 1.31 x10^6, (H) 2.62x10^6 genomes/mL. (B) corresponds to the LOD of the scFV3+3 SERS assay; (C)-(H) correspond to 2x, 4x, 8x, 16x, 32x, and 64x the SERS LOD. Arrows indicate visible bands at the sample line.

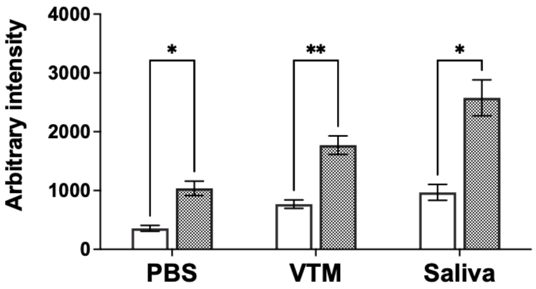

Figure S9. Comparison of scFv3+3 SERS assays carried out in PBS, VTM, and saliva. 50 ng of Wuhan-Hu-1 trimeric spike protein was diluted in each buffer and the assay completed according to the Experimental Section. White and shaded bars represent assays carried out in the absence and presence of antigen, respectively. For statistical analysis, two-way ANOVA followed by Šidák's multiple comparisons test was performed: ns = not significant. *: $p<0.05$; **: $p<0.01$.
